# Performance of family planning clinics in conducting recommended HIV counseling and testing in Mombasa County, Kenya: a cross-sectional study

**DOI:** 10.1186/s12913-019-4519-x

**Published:** 2019-09-14

**Authors:** McKenna C. Eastment, George Wanje, Barbra A. Richardson, Faiza Nassir, Emily Mwaringa, Ruanne V. Barnabas, Kenneth Sherr, Kishorchandra Mandaliya, Walter Jaoko, R. Scott McClelland

**Affiliations:** 1 Department of Medicine, University of Washington, Seattle, WA USA; 2 Department of Global Health, University of Washington, Seattle, WA USA; 3 Department of Biostatistics, University of Washington, Seattle, WA USA; 4 Fred Hutchinson Cancer Research Center, Vaccine and Infectious Disease Division, Seattle, WA USA; 5 Department of Obstetrics and Gynecology, Coast General Hospital, Mombasa, Kenya; 6 Mombasa County Department of Health, Mombasa, Kenya; 7 Department of Epidemiology, Seattle, WA USA; 8 University of Nairobi, Medical Microbiology, Nairobi, Kenya

**Keywords:** HIV testing, Family planning clinics

## Abstract

**Background:**

A high proportion of African women utilize family planning (FP) services. Accordingly, incorporating HIV testing into FP services may strategically target the first WHO 90–90-90 goal of 90% of people living with HIV knowing their status.

**Methods:**

The objective of this analysis was to determine the proportion of new FP clients counseled and tested for HIV, as well as correlates of HIV testing, in a random sample of 58 FP clinics in Mombasa County, Kenya. Structured interviews of FP clinic managers collected data on characteristics of FP clinics and staff. Study staff performed a 3-month review of FP registers, summarizing new client HIV testing and counseling (HTC). Because overall rates of HTC were quite low, a binary variable was created comparing clinics performing any HIV counseling and/or testing to clinics performing none. Generalized linear models were used to calculate prevalence ratios (PR) and identify correlates of HTC. Factors associated with any HTC with a *p*-value < 0.10 in univariate analysis were included in a multivariate analysis.

**Results:**

Of the 58 FP clinics, 26 (45%) performed any counseling for HIV testing, and 23 (40%) performed any HIV testing. Counseling for HIV testing was conducted for 815/4389 (19%) new clients, and HIV testing was performed for 420/4389 (10%). Clinics without trained HIV testing providers uniformly did not conduct HIV counseling and/or testing (0/12 [0%]), while 27/46 (59%) of clinics with ≥1 provider performed some HTC (*p* < 0.001). In the subset of 46 clinics with ≥1 trained HIV testing provider, correlates of performing HTC included being a public versus non-public clinic (PR 1.70 95%CI 1.01–2.88), and having an HIV comprehensive care center (CCC) onsite (PR 2.05, 95%CI 1.04–4.06).

**Conclusion:**

Trained HIV testing providers are crucial for FP clinics to perform any HTC. Approaches are needed to increase routine HTC in FP clinics including staffing changes and/or linkages with other testing services (in standalone VCT services or lab facilities) in order to improve the implementation of existing national guidelines. A future cluster randomized trial is planned to test an implementation strategy, the Systems Analysis and Improvement Approach (SAIA) to increase HTC in FP clinics.

## Background

There is a large burden of human immunodeficiency virus (HIV) in African women of childbearing age [1–3]. In 2016, there were an estimated 4500 new HIV infections in adults per day globally, of which 43% were in women, and the majority of these new infections occurred in sub-Saharan Africa [3]. To target the World Health Organization’s first 90–90-90 goal of having 90% of people with HIV knowing their status, improvements in the implementation of HIV testing and counseling (HTC) are needed. Many women in sub-Saharan African access family planning (FP) services [4–7], including 44% of women in Mombasa County, Kenya. As women that are likely sexually active and potentially at risk for HIV infection, this is a population that should be screened for HIV [8]. Integrating HIV testing and prevention with FP services is a promising approach to optimizing healthcare delivery, and is supported by the WHO’s Global Health Sector Strategy on HIV 2016–2021 [9, 10].

There is limited literature on the implementation of integration of HIV testing into FP services. One study in Tanzania reported that 45% of FP clinics surveyed were ready to integrate these two services [11]. Another study in Uganda examined integrating HIV testing and FP services delivered by community health workers, but not in FP clinics [12]. Two studies have investigated different integration strategies aimed at increasing HIV testing in FP clinics in Kenya. These studies showed mixed results, but generally favoring integration of HIV testing in FP clinics in one form or another. One illustrated that an FP provider training intervention increased HIV testing in FP clinics [13]. The second study in Kenya tested four different approaches to integrating HIV testing and FP services and found that only high cumulative exposure to integrated HTC and FP care led to a significant increase in HTC after adjustment for potential confounding factors [14]. Understanding current practices, as well as factors associated with successful integration of services, will be important for strengthening existing linkages between HIV testing and FP services globally.

Mombasa County is located on the Indian Ocean coast of Kenya, with a population of approximately 1.1 million [15]. Mombasa County’s adult (aged 15–49 years) HIV prevalence is estimated at 7.4%, with a higher 10.5% prevalence in women [15, 16]. Approximately 44% of women of reproductive age access family planning services in 132 FP clinics across the county [4]. Family planning clinics can be free-standing clinics or be part of a larger facility with other clinic types (antenatal care, HIV clinic, etc). Family planning clinics are generally provided with FP commodities at no cost from the Ministry of Health, regardless of whether they are public or private facilities. Public facilities provide FP commodities and HIV testing at no cost to clients, while private facilities typically charge a convenience fee. Kenya’s National AIDS and STI Control Program (NASCOP) mandates HIV testing in FP clinics, stating that “Failure to offer HIV testing and counseling is unacceptable, and will be considered negligent” [17]. Records for FP clients are maintained in paper-based FP registries that document client characteristics, FP commodities dispensed, and beginning in 2008, columns for HIV counseling conducted and HIV testing results. HIV counseling and testing can be either provider-initiated or client-initiated in these FP clinics, but at the time of this survey, there was little HIV testing and most was provider-initiated [18]. Screening HIV testing in Kenya is often rapid, point-of-care testing with whole blood. A confirmatory HIV test is performed to confirm all positive screening results per the national HIV testing algorithm. If there are discordant results between the initial screening test and the confirmatory test, clients are referred to the laboratory for further venous blood testing. Training through NASCOP or other medical regulatory bodies is required for all providers performing HTC; annual hands-on refresher training is also recommended [18].

Despite the strong recommendation and rationale for HTC to be performed in women accessing FP services, data on current HTC practices in FP clinics in Kenya are sparse. The Mombasa County Department of Health sought a better understanding of current HTC practices in their FP clinics. The present study quantified HTC in FP clinics, and identified both facility-level and clinic manager-level correlates associated with clinics’ performance of HTC in a high HIV prevalence setting in Kenya.

## Methods

### Data collection

A cross-sectional study was conducted in a random sample of 60 (45%) FP clinics out of 132 FP clinics operating in Mombasa County in 2016. Two of the selected clinics were found to have no ongoing FP clinic activities, and were excluded from further analyses. The sample size was chosen to sample approximately half of FP clinics in Mombasa County. Study staff visited FP clinics and scheduled meetings with clinic managers. These meetings occurred on the same day as the visit if clinic managers were available. If clinic managers were unavailable, then the meeting was scheduled for a subsequent day. Brief structured interviews with FP clinics managers collected information about each clinic including size, location (urban, peri-urban, rural), size of population served, public versus private facility, presence of academic or non-governmental organization (NGO) support, what other clinics are co-located with the FP clinic, if the FP clinic holds regular management meetings, and clinic staff characteristics (number and type of staff, experience and education level of clinic manager, number of providers trained in HIV testing and counseling, and whether the clinic manager is aware of the current Kenyan National HIV guidelines). These interview questions were modelled after questions used successfully in the original SAIA trial [19]. Following the interview, study staff performed an evaluation of HIV testing commodities (HIV testing kits, alcohol, cotton, lancets), to determine if they were in stock and not expired. Finally, staff abstracted data from the FP clinic register for the complete 3-month period preceding the date of data abstraction. The most recent complete and consecutive months were used for this analysis. Register pages were also examined for any duplicate entries. Digital photographs were then taken of the corresponding register pages with names masked. Study staff used these images to abstract data including number of new FP clients, number of new clients counselled for HIV testing, number of new clients tested for HIV, number newly positive for HIV, and referral processes (referred to another health facility, and reasons for referral). Over an 8-month period, multiple stakeholder meetings and results dissemination forums occurred with different members of the Mombasa County Health Management Team, sub-County Medical Officers of Health, reproductive health coordinators, family planning coordinators, the Chief Officer of Public Health, Chief Nursing Officer, and Director of Reproductive Health, to discuss this study and define HTC best practices. Data quality and interpretation was discussed at these meetings, including the handling of missing data in the registers. There was a broad consensus agreement that missing information on HIV counseling and testing in the registers indicated that these services were not performed. Data from the register photographs were initially recorded on paper, then entered manually into a REDCap database [20]. All data collection forms were reviewed for any missing responses, and addended as needed. In addition, FP register data abstraction forms were reviewed by a second study team member who independently viewed the digital images of the FP record, verifying that the same responses were generated by both study team members. Any discrepancies identified between data abstraction by different study team members were resolved with consensus agreement of the staff. Following data entry, study staff performed a question by question assessment (line-listing) comparing hard copy data collection forms to the digital database to identify and correct any key-in errors.

### Data analysis

Because HTC was performed infrequently in the vast majority of FP clinics, any HIV counseling or testing was compared to no HIV counseling or testing. Generalized linear models with a log link and binomial family were used to calculate prevalence ratios (PR) and identify correlates of HTC. Factors associated with HTC with a *p*-value < 0.10 in univariate analyses were included in a multivariate analysis [21]. Additional analyses examined correlates using a multiplicative performance score (proportion counseled x proportion tested) and an additive performance score (proportion counseled + proportion tested). All analyses were conducted using Stata version 15 (College Station, Texas) [22].

This study was conducted in close collaboration with the Mombasa County Department of Health. Prior to data collection, several meetings were held with sub-County Medical Officers of Health to incorporate their input into development of the study procedures. This study was approved by the Human Subjects Research Committee of the University of Washington (STUDY00001851), the Kenyatta National Hospital-University of Nairobi Ethics Research Committee, and Mombasa County.

## Results

Baseline characteristics of the 58 FP clinics included in this analysis are presented in Table 1. There were 14 (24%) urban and 44 (76%) non-urban (a mixture of rural and peri-urban) FP clinics. The median size of clinic staff was 4 providers (interquartile range [IQR] 2–7), with a median of 2 providers trained in HIV counseling and testing (IQR 1–3). The majority of FP clinic managers had obtained at least a post-secondary diploma (*n* = 45, 78%). Most clinic managers were aware of the Kenyan National HIV guidelines (*n* = 49, 84%), and 14 (29%) managers could show the guidelines to study staff. Over half of clinics were private (*n* = 32, 55%). Only 29 (50%) clinics had HIV test kits available for HIV testing, and these were primarily Determine™ HIV-1/2 Ag/Ab Rapid Test (*n* = 26).

**Table 1:** Descriptive characteristics of 58 family planning clinics

Variable	N (%) or Median (IQR)
Clinic staff size	4 (2–7)
Number of providers trained in HIV counseling and testing	2 (1–3)
Clinic manager is aware of current Kenyan National HIV guidelines	49 (84)
Clinic manager able to produce current HIV guidelines	14 (29)
Public facility	24 (41)
Urban clinic location	14 (24)
Presence of NGO support	35 (60)
Facility also has onsite HIV CCC	30 (52)
Regular FP clinic management meetings held	38 (66)
Number of all clients (new and return) seen in 3 months	
≤ 200 clients	34 (59)
201–400 clients	17 (29)
> 400 clients	7 (12)

Abbreviations: *IQR* Interquartile range, *HIV* Human immunodeficiency virus, *NGO* Non-governmental organization, *CCC* Comprehensive care center, *FP* Family planning

In the 58 clinics, there were 4583 new FP clients seen during the 3-month data abstraction interval. The majority of FP register data regarding HIV counseling and HIV testing were missing, with 3335 (73%) new FP clients missing counselling information, and 3336 (73%) missing HIV testing information. Only 26 (45%) clinics recorded any counseling for HIV testing, and only 23 (40%) clinics recorded any HIV testing. In the 3-month periods surveyed, 814 (18%) new FP clients were counseled for HIV testing and 419 (9%) tested for HIV. There were 8 (2%) new HIV diagnoses in the 3-month periods surveyed across all clinics. The majority (*n* = 538, 66%) of HIV counseling was performed at only 4 FP clinics and the majority (*n* = 302, 72%) of HIV testing was performed at only 3 FP clinics.

Clinics without staff trained to counsel and test for HIV uniformly did not conduct HIV counseling and/or testing (0/12 [0%]), while 27/46 (59%) of clinics with ≥1 provider performed some HTC (*p* < 0.001). Additional analyses of the clinic-level correlates of performing HIV counseling and testing were conducted in the subset of 46 clinics with ≥1 trained HIV testing provider (Table 2). Univariate correlates of performing any HTC included being a public versus non-public clinic (PR 1.70 95% confidence interval [CI] 1.01–2.88), and having an HIV comprehensive care center (CCC) onsite (PR 2.05, 95%CI 1.04–4.06). These associations were weaker, and no longer statistically significant, in multivariate analyses (public versus non-public aPR 1.37 95%CI 0.79–2.39; onsite CCC aPR 1.44 95%CI 0.62–3.36). Furthermore, clinics that were performing HTC were 50% more likely to have a clinic manager aware of the national HIV testing guidelines (PR 1.52, 95%CI 0.51–4.58), were 80% more likely to be urban (PR 1.80, 95%CI 0.80–4.09), and were almost twice as likely to hold routine management meetings (PR 1.93, 95% CI 0.92–4.04), though these did not reach statistical significance.

**Table 2:** Family planning clinic and clinic manager characteristics associated with any HIV counseling and/or testing (HTC) in 46 FP clinics with at least 1 staff trained for HTC in Mombasa County, Kenya

Variable (exposure)	Any HIV counseling and/or testing n (%)	No HIV counseling and/or testing n (%)	Unadjusted PR (95% CI)	*p*-value	Adjusted PR^b^ (95% CI)	*p*-value
Number of clinic staff (continuous)	5 (2–14)^a^	5 (2–14)^a^	1.00 (0.98, 1.03)	0.7		
Clinic manager is aware of current Kenyan National HIV guidelines	25 (93)	24 (77)	1.52 (0.51, 4.58)	0.5		
Public FP clinic compared to non-public FP clinic	17 (63)	7 (23)	1.70 (1.01, 2.88)	0.048	1.37 (0.79, 2.39)	0.3
Urban clinic location (as compared to non-urban)	23 (85)	21 (68)	1.80 (0.80, 4.09)	0.2		
Presence of NGO support	17 (55)	18 (58)	1.09 (0.66, 1.82)	0.7		
Number of all clients (new and return) seen in 3 months ≤200 as referent						
	14 (52)	20 (65)				
201–400 clients	9 (33)	8 (26)	1.34 (0.80, 2.23)	0.3		
> 400 clients	4 (15)	3 (10)	1.29 (0.66, 2.52)	0.5		
Facility also has onsite HIV CCC	21 (78)	9 (29)	2.05 (1.04, 4.06)	0.039	1.44 (0.62, 3.36)	0.4
Regular FP clinic management meetings held	22 (81)	16 (52)	1.93 (0.92, 4.04)	0.08	1.51 (0.68, 3.37)	0.3

Abbreviations: *FP* Family planning, *PR* Prevalence ratio, *CI* Confidence interval, *NGO* Non-governmental organization, *CCC* Comprehensive care center

^a^Presented as median and interquartile range

^b^Adjusted for public clinic vs non-public clinic, presence of CCC, and/or regular management meetings

## Discussion

This cross-sectional study aimed to quantify HTC in FP clinics, and to understand facility-level and manager-level correlates of performing any HTC. Less than half of FP clinics are performing any counseling or testing for HIV. Those clinics that are performing some testing are not reaching a significant number/proportion of FP clients. This study highlighted the necessity of having FP clinic providers trained in HTC. In those clinics with at least one HTC provider, public facilities as compared to private facilities, and facilities with an onsite CCC were associated with HTC. Ultimately, only 18% of new FP clients were counseled about HIV testing and only 9% were tested. Less than half of the clinics surveyed recorded any HIV counseling or testing.

The idea of integrating HIV testing with FP services is supported in the literature as a promising approach to reaching the first WHO 90–90-90 goal of 90% of people knowing their HIV status [23–25]. However, there is little literature on current HTC practices in FP clinics. One study from Tanzania found that only 45% of health facilities were ready to integrate HIV testing into FP clinics [11]. Determinants of integration readiness were similar to the results in this analysis, including in-service training of staff and being government-owned. Other determinants included routine management meetings, availability of guidelines, and availability of laboratories for HIV testing. The proportion of FP clients counseled (18%) and tested (9%) for HIV in this analysis yielded 8 (2%) new HIV diagnoses. This 2% is similar to a nationwide campaign in 2017 and 2018 in Kenya that tested approximately 13 million people for HIV, yielding 1.4% new HIV diagnoses [26]. This is in the context of the adult HIV prevalence in women in Mombasa County at 10.5% [15, 16]. Ultimately, integrating HTC into FP could provide an important approach for identifying HIV-positive women that might otherwise remain undiagnosed, but needs a better performing system.

This study had some notable strengths. Data from both interviews and review of FP registers were collected from a range of clinic types and locations across Mombasa County. To address the priorities of Mombasa County, this research was conducted in close collaboration with the Department of Health. This effective partnership will facilitate implementation of changes to improve HTC performance in FP clinics in the next stage of this research. In addition, these data were interpreted in collaboration with co-investigators from the County, who are familiar with the FP clinics and registers. There was 100% participation from the selected FP clinics, which represented a random sample of almost half of FP clinics in the county.

This study also had a number of limitations. First, data from FP registers had a large amount of missing entries. It is possible that HTC was being performed in the FP clinic but not documented appropriately. Our collaborators from Mombasa County and FP clinic staff advised that missing entries should be considered to represent HIV counseling and testing not performed, but there is no way to independently verify this. Second, HTC may have been performed in another clinic (e.g. in an attached CCC, voluntary counseling and testing site, or antenatal care clinic) and not documented in the FP register. This topic will be explored in future in-depth interviews. Third, self-report from clinic managers could be subject to bias (for example, are regular management meetings being held?). If these were over-reported compared to actual practice, the true effect of management meetings on HTC performance could have been underestimated. Whenever possible, study staff attempted to verify reported data. For example, they routinely asked to examine HIV testing supplies to confirm that they were in stock and not expired. Fourth, the three-month duration of data collection may have missed periodic or annual variations in HTC. Fifth, there were likely client-level barriers and facilitators to HTC, but these were not assessed in this study. The focus was on clinic-level factors that could be addressed with quality improvement exercises. Finally, there was a modest sample size, particularly in the correlates analysis that was restricted to only 46 clinics with a trained HTC provider. While important correlates were identified with this sample size, weaker associations may have been missed.

## Conclusions

In conclusion, HIV testing was being conducted in approximately 40% of FP clinics in Mombasa County, despite strong national Kenyan guidelines recommending HIV testing in all FP clients. The high number of new FP clients being seen in FP clinics argues for the great potential to reach more women for HIV counseling and testing in these settings. While Kenyan national guidelines provide a strongly worded recommendation to counsel and test all FP clients for HIV, and County leadership is supportive of these national guidelines, improved implementation at the individual clinic level is warranted. Furthermore, performance data on HTC are collected monthly, reviewed by County officials, and discussed with individual FP clinics. Nonetheless, HTC remains low, suggesting a need for additional quality improvement efforts to catalyze increases in HTC. Because trained HIV testing providers are crucial for FP clinics to perform any HTC, mechanisms are needed to increase HTC providers in FP clinics or to link these clinics with voluntary counseling and testing sites or laboratory facilities able to perform testing. For FP clinics that are not co-located with HIV care clinics, improving linkages to external HIV care clinics could also facilitate HTC. These strategies have the potential to propel countries closer to the first WHO 90–90-90 goal.

## Data Availability

The datasets used and/or analysed during the current study are available from the corresponding author on reasonable request and upon approval from the Kenyatta National Hospital-University of Nairobi Ethics Research Committee.

## Abbreviations

CCC: Comprehensive care center
FP: Family planning
HIV: Human immunodeficiency virus
HTC: HIV counseling and testing
IQR: Interquartile range
NASCOP: National AIDS and STI Control Program
PR: Prevalence ratios
VCT: Voluntary counseling and testing
WHO: World Health Organization
